# Supplementary material for: The impact of donor transition on continuity of maternal and newborn health service delivery in Rwenzori sub-region of Uganda: a qualitative country case study analysis
Source: Global Health. 2023 Jul 10;19:48. doi: 10.1186/s12992-023-00945-6 (PMC10334577; doi:10.1186/s12992-023-00945-6)
Supplement: Supplementary file 1 — Supplementary Material 1 [file 12992_2023_945_MOESM1_ESM.docx]

**The impact of donor transition on continuity of maternal and newborn health service delivery in Rwenzori sub-region of Uganda: A qualitative country case study analysis.**

*Eric Ssegujja^1*^, Justine Namakula^1^, Allen Kabagenyi^2^, Carol Kyozira^3^, Timothy Musila^3^, Henry Zakumumpa^1^, Freddie Ssengooba^1^*

^1^ Department of Health Policy Planning and Management, School of Public Health, College of Health Sciences, Makerere University- Kampala, Uganda.

^2^Department of Statistics and Population Studies, College of Business and Management Studies, Makerere University- Kampala, Uganda.

^3^Ministry of Health- Kampala, Uganda

*Corresponding author: [ssega2001@yahoo.com](mailto:ssega2001@yahoo.com)

**Abstract**

**Background**

The transition of donor-supported health programmes to country ownership is gaining increasing attention due to reduced development assistance for health globally. It is further accelerated by the ineligibility of previously Low-Income Countries’ elevation into Middle-income status. Despite the increased attention, little is known about the long-term impact of this transition on the continuity of maternal and child health service provision. Hence, we conducted this study to explore the impact of donor transition on the continuity of maternal and newborn health service provision at the sub-national level in Uganda between 2012-2021.

**Methods**

We conducted a qualitative case study of the Rwenzori sub-region in mid-western Uganda which benefited from a USAID project to reduce maternal and newborn deaths between 2012 and 2016. We purposively sampled three districts. Data were collected between January and May 2022 among subnational key informants (n=26), national level key informants at the Ministry of Health (3), national level donor representatives (3) and subnational level donor representatives (4) giving a total of 36 respondents. Thematic analysis was deductively conducted with findings structured along the WHO's health systems building blocks (*Governance, Human resources, Health financing, Health information systems, medical products and supplies and service delivery*) framework.

**Results**

Overall, continuity of maternal and newborn health service provision was to a greater extent maintained post-donor support. The process was characterised by a phased implementation approach. The embedded learning offered the opportunity to plough back lessons into intervention modification which reflected contextual adaptation. The availability of successor grants from other donors (such as Belgian ENABEL), counterpart funding from the government to bridge the gaps left behind, absorption of USAID-project salaried workforce (such as midwives) onto the public sector payroll, harmonisation of salary structures, the continued use of infrastructure (such as newborn intensive care units), and support for MCH services under PEPFAR support post-transition contributed to the maintenance of coverage. The demand creation for MCH services pre-transition ensured patient demand post-transition. Challenges to the maintenance of coverage were drug stockouts and sustainability of the private sector component among others.

**Conclusion**

A general perception of the continuity of maternal and newborn health service provision post-donor transition was observed with internal (government counterpart funding) and external enablers (successor donor funding) contributing to this performance. Opportunities for the continuity of maternal and newborn service delivery performance post-transition exist when harnessed well within the prevailing context. The ability to learn and adapt, the presence of government counterpart funding and commitment to carry on with implementation were major ingredients signalling a crucial role of government in the continuity of service provision post-transition.

***Keywords:*** Donor transition, maternal and child health, sustainability, coverage maintenance.

**Background**

The transition from external funding for health programs in low and middle-income countries has gained increased importance in global health and development discourse in recent years (1, 2). It is at the convergence between improvements in economic status and health indicators in some developing countries that had previously benefited from Global Health Initiatives (GHI)(3). It is also at the intersection with the current trends in global health financing which aim to increase country ownership and strengthen local capacities (4). Different forms of donor exit strategies include financial (on-budget and off-budget support), technical, onsite and offsite support have been documented. However, irrespective of the form that this cessation takes, their effect on the sustainability and maintenance of service delivery gains achieved has been reported to take different forms such as a relapse, continuity or even better performance thereafter (5). Uganda has enjoyed relatively consistent donor support for reproductive maternal newborn and child health (RMNCH) programming right from the 1978 Alma Ata declaration of Primary Health Care (PHC), through the Safe Motherhood program in the 1990s, up to the 2000 initiation of the Millennium Development Goals (MDGs) and currently during the Sustainable Development Goals (SDG). By donor transition, we refer to a systematic cessation of financial and technical support from bilateral and multilateral donors for Global Health Initiatives (GHI) to central support by respective governments.

Drawing from earlier case studies across the globe, the inability of donor-recipient governments to sustain the scale of intervention implementation post-transition has prompted a re-think in transition prioritisation and planning(6). Studies have revealed that despite the capacity building during donor support, the sustainability of public health gains remains a big challenge after the cessation of external assistance (7, 8). There are wide perceptions that transition arrangements are either absent, poorly planned or generalised as sustainability or exit strategies which have led to the collapse of intervention gains upon donor exit(8, 9). The same predicament was anticipated for RMNCH interventions and yet most evaluations of such interventions have been conducted immediately after the program end date without a long-term focus hence being unable to determine the extent of impact in the longer term.

Towards the end of the MDG era in 2011, Uganda received substantial financial support for the improvement of the RMNCH service delivery. The United States Government’s President’s Global Health Initiative on Women and Girls was an integrated response to global health challenges(10). In Uganda, implementation was supported by United States Agency for International Development (USAID) and the Centre for Disease Control and Prevention (CDC)(11). It included proposed strategies which aimed at strengthening the health systems' capacities using system thinking to address major bottlenecks associated with maternal deaths(12). The process of preparing the country to take over was further strengthened through the involvement of other in-country RMNCH partners including the private health sector.

**USAID’s Saving Mothers Giving Life (SMGL) Intervention**

The Saving Mothers Giving Life (SMGL) program was launched in 2012 and first piloted in four districts in Rwenzori perceived to have shouldered the highest burdens of maternal mortality in Uganda at the time(4). The goal was to support countries in reducing maternal deaths by up to 50% within one year in accelerated progress towards the MDG 5 target of a 75% reduction in Maternal Mortality ratio (MMR) by 2015 (13). It targeted countries where the US had a significant global health investment and presence which included Zambia and Uganda(10). Its design was informed by current trends in global health financing which aim to increase country ownership and strengthen local capacity through strengthening existing public health systems, particularly at the sub-national level(4). The intervention focused on the ‘three delays’ model which focuses on the critical window during labour, delivery and the first 24-48 hours after delivery (10). The intervention approach had a strong health system-strengthening ethos at subnational levels while ensuring it served to strengthen existing public-private health service provider networks as reflected in table 1 below. It was anticipated to demonstrate a significant reduction in maternal and newborn deaths in Uganda (4).

**Table 1: Programmatic components along the three delay model**

| **Delay one (decision to seek care)** | **Delay two (timely access to health care)** | **Delay three (quality healthcare)** |
| --- | --- | --- |
| ***Strategy 1;*** *community engagement and empowerment for maternal and new born health improvement* | ***Strategy 1;*** *decrease distance to skilled birth attendance by increasing the number of EmONC facilities* | ***Strategy 1;*** *Ensure facilities have adequate infrastructure for EmONC*  ***Strategy 2;*** *Ensure adequate medical supplies, equipment and medication* |
| ***Strategy 2;*** *increased birth preparedness, demand for facility deliveries, use of preventive health services* | ***Strategy 2;*** *improve accessibility of EmONC services* | ***Strategy 3;*** *Ensure sufficient trained health workers to provide care*  ***Strategy 4;*** *Improve quality of care and ensure its evidence-based.*  ***Strategy 5;*** *ensure referral capacity exists*  ***Strategy 6;*** *Support effective maternal and perinatal health surveillance.* |

Although previous studies have evaluated the impact of the SMGL intervention in Uganda, these were conducted during the implementation of the intervention or immediately after completion(4, 11, 12). Reports of increased facility attendance, reduced delays in accessing health services, improvements in quality of services offered were some of the key performance improvement highlights(12). There is a paucity of research examining the medium to long-term sustainment of maternal and newborn health outcomes registered during the SMGL intervention period (2012-2017) Rwenzori region. The health system, political and financing factors which influenced whether the RMNCH gains registered were sustained remain poorly understood despite its public health importance in Uganda and other similar settings. Hence this study was conducted to explore the impact of donor transition on the continuity of maternal and new born health service provision at the sub-national level in Uganda between 2012-2021

**Methods**

***Study design***

We report qualitative findings from a larger mixed-methods study conducted in the Rwenzori sub-region in mid-western Uganda where the SMGL project was implemented (11). The quantitative arm of the study examined trends in selected MNCH indicators (such as maternal and newborn mortality, and institutional deliveries) based on secondary analysis of DHIS-2 data over 10 years (between 2012 and 2021). In this paper, we qualitatively explored the health system factors according to the WHO building blocks which may have contributed to the continuity of maternal and new born health service provision at the sub-national level in Uganda between 2012-2021 following donor transition in the Rwenzori region in Uganda.

***Study context***

Uganda is a beneficiary of multiple Global Health Initiatives supporting improvement in maternal, newborn and child health services delivery. Through the 1990’s the dominant player was the Safe Motherhood program born out of the 1985 landmark Lancet publication “Maternal Mortality a neglected Tragedy: Where is the M in MCH” and the subsequent Nairobi Conference. It had a great impact on national MCH policy and service delivery(14). The country joined global efforts in translating the aspirations of the 2000 Millennium Summit which ushered in the Millenium Development Goals (MDGs) with a specific focus on MDG 4 of reducing under-5 mortality by two-thirds and MDG 5 of reducing Maternal Mortality Rate (MMR) by three-quarters by 2015 (15, 16). These were localised into national MCH policy and programming. Despite this support, the country still suffers from substantially high maternal and newborn mortality rates with chronic health systems challenges such as timely and quality care partly accounting for the failure to achieve global and national targets(10, 11, 13). It is within this context that the United States Government funding through the Saving Mothers Giving Life (SMGL) project was implemented in Uganda with the pilot phase happening between 2012-2017 and scale-up from 2016-2021(10, 11, 13). Uganda was selected as the implementation site because it was identified as one of the Sub Sahara African countries with the highest maternal mortality and the Rwenzori region was specifically selected because it had the highest burden of maternal mortality in Uganda at the time. The transition timelines referred to in this study will denote a period from 2017 up to when the funding ended in 2022.

***Figure 1: Timelines for project implementation and transition period***


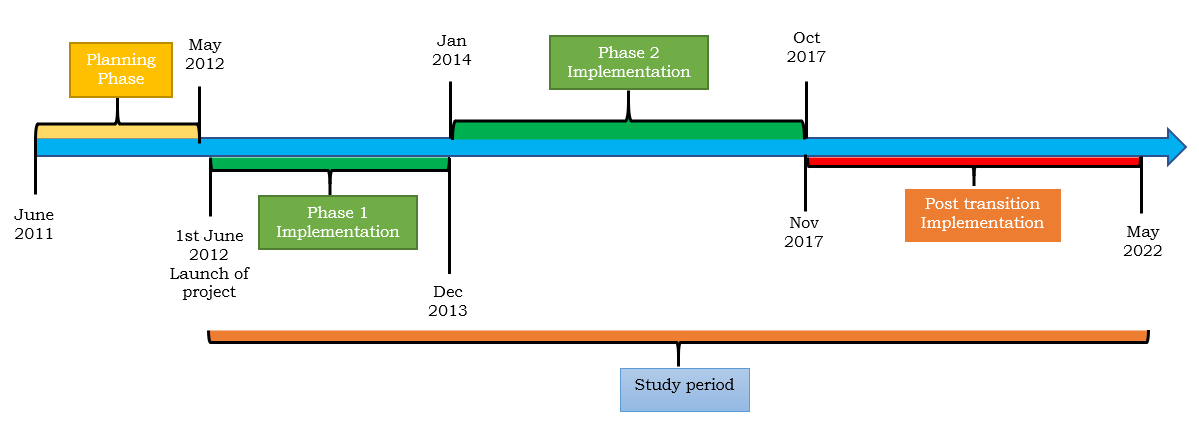


***Study sites and sample selection***

We conducted this study in the Rwenzori sub-region located in Mid-Western Uganda where the SMGL project was implemented. We purposively selected three districts of Kabarole, Kamwenge and Kyenjojo (10, 13).

***Data collection***

*Document review*

We commenced with a desk review of key documents relating to the implementation of the SMGL intervention and analysis of secondary data and records from the Ministry of Health and project-related reports guided by the study objectives. Emerging insights from the qualitative data collection were further triangulated by a targeted review of documents such as key project reports, Ministry of Health annual reports, related MCH policies and guidelines as well as published project work and any other grey literature from online sources.

*Semi-structured In-depth interviews*

This was followed by qualitative data collection by the two authors (ES and HZ) who are well-experienced in qualitative research methods and familiar with national-level MCH policy processes. The data collection tools were specifically developed for this study. Informed by the emerging information from the document review and study objectives, the tools explored the factors that may have contributed to the continuity of service delivery post donor transition according to respondent’s perspectives. The activity was conducted between January and May 2022. Prior, contacts were initiated with the district health managers about the study aims and procedures. The list of potential respondents together with contacts was compiled with the help of district officials. These were then contacted and informed about the possibility of participating in the study. The ones who agreed, a day and time to conduct the interview was agreed. No further contact was made with potential respondents who indicated unavailability to participate. Overall, none among those who initially expressed willingness to participate was not interviewed. On the day of the interview, a secure and quiet place was identified from where verbal or written consent was obtained before conducting the interview. Respondents were asked guide questions which broadly covered their experiences with implementing the SMGL project, variations in service delivery during and after implementation of the project and reasons behind the observed performance of key MCH indicators post-transition. Interviews were recorded using digital audio recorders with field notes taken during and after the interviews which were shared at the end of each field day during the daily debrief meetings where further notes were taken using notebooks. Overall, the total sample comprised of subnational key informants (n=26), national level key informants at the Ministry of Health (3), national level donor representatives (3) and subnational level donor representatives (4) giving a total of 36 respondents for the study.

***Analytical framework;***

We adopted the World Health Organisation (WHO) health systems building blocks framework(17). It consists of six building blocks which include; Governance, Human resources for Health (HRH), Service delivery, Health Management Information systems (HMIS), Health Financing and Medical products and supplies. It is a health systems assessment framework which allows for the analysis of the entire health systems performance diving deep into its constituent blocks. Its proponent is the World Health Organisation and it has been applied in several contexts to analyze the health systems performance. It is however lean on articulating demand side/ community components and the dynamic interactions across the building blocks which have potential to impact the health systems performance. We conceptualised the Governance to denote aspects of project capacity building directed towards strengthening the oversight and accountability functions of the health systems. Human resources for Health (HRH) looked at aspects of skills and other capacity building targeted toward the health workforce. Service delivery on the other hand looked at inputs that went into ensuring that the quality of services was of the recommended standards. Health Management Information systems (HMIS) focused on data and information systems strengthening while, Health Financing focussed on the funding aspects. Finally, Medical products and supplies block examined the capacity strengthening that went into ensuring that medicines and other supplies were at all times present within the health systems.

***Data analysis***

All interviews were conducted in English and hence transcribed verbatim into Microsoft office word. The coding was conducted in Atlas. ti, (ver 6) where all transcripts were imported. The codebook development was informed by the health systems building blocks, the applied framework which informed the design and overall methodology for the study. A deductive coding process was adopted. Hence the team used six codes comprising the health systems building blocks; Governance, Human resources for health (HRH), Finance, Health Management Information systems (HMIS), service delivery, Medical products and supplies. Chunks of text from the transcript would be highlighted and attached to the corresponding code after being scrutinised. Two authors (ES & JN) independently coded five transcripts to enhance reliability in the coding process. Thereafter, the two researchers discussed and reached a consensus on text content after resolving inconsistencies in the application of codes. They then independently coded the remaining transcripts with the ongoing discussion regarding the application and comparison of text using a deductive approach. Query reports were run and a manual pile sorting exercise followed where chunks of text were again read through and organised according to the emerging underlying meaning which formed the themes and subthemes using a thematic data analysis technique. Selected representative quotations to bring out the respondent’s voices have been used while writing up the results. Respondents’ identities have been protected through anonymised pseudonyms as codes reflective of a combination of respondent category, position or title at the time of the interview and the sequenced interview number during the data collection process.

**Results**

The results are presented according to emerging implementation dynamics that characterised the duration of project implementation experiences in Uganda over ten years (2012-2022). It was implemented in sequences with the 2012-2013 covering phase one, a proof of concept stage. Phase two ran between 2014-2017 and aimed to consolidate lessons that had worked well while building district capacities to transition(10). Between 2012-2017 within the Rwenzori region, implementation was facilitated by Baylor Uganda Limited, USAID Applying Science to Strengthen and Improve Systems (ASSIST) and other implementing partners. It is important to note that while the results are separated by building blocks, attention has been paid to how interactions across building blocks and across different actors affect inputs, processes, outputs, and outcomes of the system which are implicitly reflected earlier on under the “analytical framework” within the methods section and later within the results (service delivery, financing) and discussion sections that follow. The summary of key emerging themes is presented in table 2 below;

**Table 2: Key results according to the health systems building blocks**

|  | **Building blocks** | **Key themes** |
| --- | --- | --- |
| 1 | Health financing | Enablers;   - Counterpart funding from government - Successor grants from other donors - Continued support from other USAID-funded projects |
|  |  | Barriers;   - High cost of servicing maternity equipment - Inability to finance some community components |
| 2 | Service delivery | Enablers;   - Demand-side component popularised services - Consistent application of learning - Heavy infrastructural upgrade - Project-procured equipment continued to serve |
|  |  | Barriers;   - Setbacks in the community referral systems - Disruptions in the private sector component |
| 3 | Human resources for health | Enablers;   - Massive absorption of project-recruited workforce - Project staff salary harmonisation with public service |
|  |  | Barriers;   - Termination of support from offsite project mentors - Some health facilities (rural) unable to retain staff |
| 4 | Health Management information systems | Enablers;   - Continued offsite support from implementing partner - Government support with reporting tools |
|  |  | Barriers;   - Data management requirements exceed capacity - Variations in capturing data within the catchment area. |
| 5 | Governance | Enablers;   - Calibre of district leadership |
|  |  | Barriers;   - Unstable district technical leadership. |
| 6 | Medical products and supplies | Enablers;   - Support for medicines and supplies from successor grants. |
|  |  | Barriers;   - Medicines and supplies stock-outs |

**Health Financing**

***Counterpart funding from the government***

Substantial commitment by the Uganda government to avail counterpart funding to bridge some of the health systems gaps post-transition was identified as a facilitator of sustainment and continuity of MNCH services. Experiences from the SMGL implementation triggered government counterpart funding which in some instances increased its budgetary support towards improving health systems support for better RMNCH quality services delivery. The SMGL theory of change was built on district systems strengthening approach to address both demand and supply side factors impeding women and newborns from getting quality RMNCH services. Having strengthened the subnational health systems, the government was able to step in and provide additional support to health systems strengthening in different aspects including the upgrade of health facilities, increased health worker salaries, recruitment of new and additional staff, increase in the wage bill and overall increased funding for maternal and child health services;

*Over time, the budgetary allocation by Government was increased to improve the health centre IIIs and health centre IV infrastructure. You might have heard about the Uganda inter-governmental funds that health centre IIs have been upgraded to HCIIIs and now it is a policy issue, you recall from 2014 that government of Uganda was not opening up new HCIIs but rather upgrading the existing HCIIs to HCIIIs. So, somehow now the health centre IIs are elevated to centre IIIs, now we have about seven of them in [the district], but at least all have received funding and it is not easy funding by the way. Now we are at a level of starting up new HCIIIs in the sub-counties. Somehow you can see the drive is now in that area, possibly an eye opener due to this project SMGL.* ***#HM_4.***

*Now the Ministry of Health through a project called Expand on Maternity Wards in H.C.IIIs and strengthen them, provides water and sanitation, bathing spaces for the mothers, provide lights, provide elements of infection control and also skill through mentorship and courses.* ***#MoH_1***

***Successor grants from other donors***

The transition period witnessed several successor grants. The Uganda Reproductive Maternal and Child Health Services Improvement Project (URMCHIP) was funded to the tune of $165 million by the World Bank, the Global Financing Facility and the Swedish Government to ensure maternal and child health programs continued to be prioritised (18). Developed in 2016 and rolled out in 2017 the URMCHIP incentivised some of the SMGL indicators through Result Based Financing (RBF). ENABEL a Belgian government-supported project implemented in the same region also prioritised support from some SMGL MCH indicators through result-based financing for select indicators. The performance indicators which were purchased under this arrangement helped to strengthen and entrench the gains achieved by sub-national units under the SMGL. These RBF projects prioritised RMNCH indicators which promoted the sustainability of gains post-transition. Other projects incentivised MCH indicators such as the PERFORM 2 SCALE project. It supported many of the aspects of quality health services which were initiated by SMGL hence leading to sustained gains. Still in Rwenzori and other regions where SMGL best practices were scaled-up. Other partners supporting MCH in the region included UNICEF and Save the Children among others.

***Continued support from other USAID-funded projects***

The US Government's interest to secure funding and support national RMNCH programming following the cessation of SMGL funding was reflected in the PEPFAR support towards integrated health systems strengthening. PEPFAR Implementing partners had a component supporting systems strengthening focussing on MCH-related indicators beyond the HIV/TB services. This extended into the period after 2021 when PEPFAR had a policy shift towards supporting local implementing partners to facilitate national health systems strengthening. Under this arrangement, Baylor Uganda Limited continued to provide offsite support within the region, particularly in aspects related to data to subnational health systems. Some of the observed maintenance of performance can be attributed to this continued support post-transition.

**Barriers**

There were some barriers which could have affected performance post-transition including the heavy burden of sustaining donor investments. In one of the districts, the cost of servicing maternity care equipment was estimated at 14 million Uganda shillings which the hospital could not afford given its resource envelope. There was another instance of servicing the ageing ambulance vehicles with over 500,000 km mileage which was perceived to be burden. The inability to fund some components post-transition such as the community component was observed. More investments in remote and rural health facilities could have affected the morale and performance of the urban health facilities which did not receive the same package of support post-transition. The unintended consequence arising out of this was that for some of the lower level urban facilities that perceived themselves as having been supported less during donor support compared to their rural peers, the motivation to uphold and continue delivery of quality services varied.

**Service delivery**

***Demand side component popularised available services***

The community demand created for MCH services including Neonatal Intensive Care Unit (NICU) during SMGL implementation still manifested post-transition. To address delay one, the project employed strategies which included; 1) strategy 1; promotion of community engagement and empowerment for improved maternal and newborn health, 2) strategy 2; increased birth preparedness, demand for facility delivery, and use of preventive healthcare services, Substantial efforts were invested towards demand creation for supported MCH services. For the new MCH services which had not been part of the standard package delivered before SMGL like NICU, the communities were made aware of the availability of these services and continued demanding and accessing them post-transition. This drew communities towards utilising those services at the health facilities as reflected in the quote below;

*‘Creating demand, because initially, they knew that such a service is not being offered on ABC, then they would also not come.’* ***#HM_5***

***Consistent application of learning from project.***

The emerging local evidence on results-based financing informed other projects supporting the purchase of RMNCH indicators which had been promoted under SMGL. The Strengthening Health Outcomes through the Private Sector (SHOPs) project implemented between 2012-2014 as part of the larger Savings Mothers, Giving Life project within the districts of Kabarole, Kamwenge, Kyenjojo and Kibale under the healthy baby voucher program had some lessons and best practices. It covered 4 Antenatal Care (ANC) visits, delivery and transport as well as postnatal care by SMGL partner Marie Stopes International through its local affiliate that served as the voucher management agency. Implementation results reflected increased public-private linkage leading to improvements and ease of referrals and collaborative arrangements in transport assistance, and increased reporting from the private sector. Part of the experiences from this would later inform the design of the Uganda Reproductive Maternal and Child Health Improvement Project (URMCHIP). Some of the key lessons learnt from the SMGL program informed the revision of national MCH guidelines and policies such as the revised Maternal and Perinatal Death Surveillance and Response (MPDSR) which would later be rolled out across the country(13). Other RMNCH indicators such as stillbirth were included in the national surveillance system with a requirement for timely notification of incidences within 24 hours. It made timely monitoring and mandated subnational and national health systems managers to investigate the causes of both maternal and perinatal deaths and address gaps within the health systems to avoid re-occurrence.

***The heavy infrastructural upgrades***

The heavy capital investment towards strengthening subnational health systems was in preparation for country ownership and take-off after 2015. Whereas building local capacities cannot solely explain the observed continuity of service delivery post-transition, respondents believed and attributed their ability to sustain the benefits to the continued use of some of the infrastructure left behind some of which was being established for the very first time within the health system such as the Neonatal Intensive Care Unit (NICU), nostalgically referred to by the communities as “the clinic for the small babies” ;

*By the way, SMGL constructed for us a maternity ward building, a theatre, and there is [also the] NICU. They demolished the [older] maternity ward which was there and they constructed a new structure. So, a lot was done, and now the picture was that the government can’t manage alone… I can say construction because if they left for us the other [old] maternity and the theatre which was there, and without NICU, I don’t think the government would have constructed those.* ***#HW_1***

*[they] really left a lot of things on the ground and they are still there, and they even have another facility which was upgraded from lower HCIIs and became HCIII because of this.* ***#HM_2***

***Project-procured equipment continued to serve***

Several medical equipment was procured during project implementation. Some of these continue to serve the purpose. These include; incubators, oxygen concentrators, Blood Pressure machines, weighing scales, baby warmers, standby generators and the fleet of ambulances for transporting expectant mothers. Timely referral which had been a chronic challenge contributing to poor maternal health outcomes was addressed through the provision of ambulances with support from donor funds. This extended after the transition from donor support and more efficiently in districts that managed to repair and provide fuel for the ambulances.

*Regarding the issue of the referral system, right now we had ambulances here because other ambulances are in the settlement but we also made sure that the Baylor ambulance was left here and is still running, we made sure that we continued repairing it so that it continues serving.* ***#HM_3***

**Barriers**

***Setbacks in the community referral systems***

The community referral systems which were initiated during the period of donor support also faced sustainability challenges post-transition. The referral systems were more supported during the period of donor support and when that ended, certain aspects could not continue particularly the voucher transport reimbursement for motorcycle riders as reflected in the quotation below;

*‘Then there was this other issue of the referral system, what they are calling community engagement, we have a very good linkage from the community to the facility, HCIIs, HCIIIs, now to HCIVs so you find the system is there working, we had boda bodas [motor cycle taxi] registered they could look for pregnant mothers and take them to the hospital and that is what we are supposed to be doing, so we knew, how many are pregnant, every mother is registered to a boda boda, and there wouldn’t be any issues of delays, so the delays stopped because there was that system, otherwise the boda bodas would take the mothers and at the end of the month they could bring the vouchers and they were reimbursed, so, there was a lot of money in play so we did several things and the system worked very well.* ***#HM_2***

***Disruptions in the private sector component.***

The program was designed with a component of improving service access through partnering with the private-for-profit maternal health service providers. This was through support for equipment improvements and subsidisation of services such as cost of conducting caesarean section. As implementation progressed, the project’s reimbursement to the private-for-profit to subsidise the cost became an incentive for them to offer c-section. The more cases they conducted, the higher the cost received from the project. As more mothers were subjected to c-section whenever they accessed delivery services from the private-for-profit providers, they came to observe that some were unnecessary and conducted against their wish. It would later become clear to them that continued access of maternal health services from the private-for-profit sector particularly delivery services would mean more deliveries by caesarean section for them. While the subsidisation introduced by the project was on, others continued to access the services but when it ceased following donor transition, many stopped seeking for delivery services from the private-for-profit maternal health service providers. This affected service utilisation from the private-for-profit providers in the medium and long term compared to how the case was when support from the project was still flowing. The reduction in volumes of delivery service affected access to quality maternal health service access which was among the main aim of the project at inception. Commenting on the cause, a respondent attributed it to the over prescription of c-section driven mainly by the incentive attached to it during project implementation as reflected in the quotation below;

*‘There was also partial misuse of this especially when it came to the private-for-profit facilities that were offering emergencies caesarean sections, yes they were supported with equipment and so on but again each Caesarean section (CS) that would be carried out in the private facility, there was an incentive, so, somehow, some of the women were subjected to CS even when it was not necessary.* ***#HM_5***

**Human resource**

***Massive absorption of project-recruited workforce.***

The ability to retain project-trained staff post-transition was another factor that was identified as an enabler of sustained coverage of services. There was a deliberate effort by the District Local Government with support from the Ministry of Health to prioritise retention and recruitment into public service project staff that had received several pieces of training and capacity building by SMGL. Attention was drawn to critical cadres especially midwives and nurses, medical doctors and anaesthetists who would operationalise Comprehensive Emergency, Obstetric and Newborn Intensive Care (CEmONIC). This worked to retain the quality of MCH services that may have contributed to the sustainability of gains post-transition as reflected in the quotation below;

*‘The good thing is government almost took up all the health workers, especially the nurses, the midwives and the clinical officers and the medical officers. For instance, our former in-charge here was recruited by Baylor in SMGL, then after the government took them all and one was transferred to Kyenjojo, so the government tried, actually SMGL helped the government because most of the health workers government took them all.’* ***#HM_10***

**Project staff salary harmonisation with public service**

Harmonisation of staff salaries between the project and public service staff served to support their retention where they were posted following their absorption into public service. An arrangement was reached between the project leadership and the Government to harmonise staff salaries. Previously, project staff were known to be paid higher salaries which made it impossible to absorb them after the project closure due to salary variations. The quotation below exemplifies this;

*The staff we trained at the end of the project said we need to level the payment. The government pay also tried to catch up a bit, so, we found that at least SMGL 2 there was no big difference between the project staff and the government staff in terms of the salary scale. So, it was very easy to tell the Ministry of Health that if you are to sustain, these staff must be paid so we tried to absorb them.* ***#HM_4***

**Barriers**

***Termination of support from offsite project mentors***

Nonetheless, some barriers to continuity of service provision were experienced post-transition. For example, some of the offsite project mentors who were Kampala-based expatriates who could not be supported in absence of funding. Whereas some retreated to Kampala others shifted to northern Uganda where SMGL was supporting wave 1 scale-up in non-SMGL districts. They could not continue offering onsite support. The more experienced peers who were early adopters within the SMGL pilot districts were identified and given more support to become champions. The aim was to have them support SMGL districts in the scale-up phase in northern Uganda. For those that were already in public service, it led to their divided attention having to support wave 1 scale-up districts and continue to offer services at their health facility of placement within the Rwenzori region.

*because those people are very expensive, getting a whole professor from [University] to come for three days in a district government cannot afford that. I think the thinking by then until now is that the region should take over the regional referral hospital. They are also not supported and they will tell you, now they are in charge of ten lower local government and they don’t have the capacity, they don’t have to human resource to do that, how many gynaecologists do they have, so that others can go to the field and then others remain. Do you have transport, do you have the money, so, by that time it was like when these people leave the regional referral hospital should take over but it couldn’t do it because it didn’t have the capacity for human resource but that was the plan,* ***#HM_4***

***Inability to retain staff by some health facilities***

Results revealed that some health facilities especially rural were unable to retain some of the recruited health workers. We came across one particular case of a Health Centre IV which had a fully equipped Neonatal Intensive Care Unit (NICU) where all trained personnel had moved on. Management had to improvise by identifying a nurse who served as the facility Mother-to-Child-Transmission (MTCT) focal person to maintain the continuity of the NICU services at the health facility. In another case, a health worker who had worked as a paediatrician could not get absorbed within the system

*for those that opted not to stay, because they had had high qualifications, people had attained higher qualifications, actually that enhancement we had a paediatrician who was working as an MO, because he had an MMed in paediatrics so he had come to do what, so when this fund wind out he decided to go.* ***#HM_3***

**Health Management information systems**

***Continued offsite support from implementing partner***

The previous implementing partner for SMGL was Baylor Uganda Limited. Immediately after SMGL ended they secured another contract from PEPFAR for HIV/TB service delivery within the same region. They continued supporting health facilities in aspects of data quality, repair of health facility RMNCH equipment and offering other offsite support. In another instance, some of the activities they engaged in were the facilitation of review meetings with the district health managers. Rather than limiting these review meetings to HIV/TB the managers extended the package to include a review of RMNCH implementation in the district. This perhaps could have facilitated the sustenance of gains post-SMGL funding

*‘Then the other thing is that at the district, we have review meetings and performance is emphasized, why are you doing this, and usually xxx[previous implementing partners] supports our review meeting, for us, we don’t only review HIV, we go beyond for maternal, neonatal, nutrition, we make it a comprehensive review, we say those are doing well we appreciate them, those are doing badly and we also identify the gaps, so we monitor performance, and then we look at the gaps, then making clear action points addressed.* ***#HM_5***

***Government support with reporting tools***

Several aspects of capacity building that may have contributed to continuity of proper data management practices post transition were mentioned by the respondents. It was reported that aspects of provision of data tools from the centre and facility management practices such as improvising through photocopying data tools, retained health worker skills of using other evidence-based data tools such as partograph among others.

*Uganda has also moved from this exercise book-based information capture. You know we were not having this health information data [tools], but those have been provided. At least now with the increase in the PHC non-wage allocation, facility and hospital management may photo copy and have adequate materials. To monitor in the labour ward, partographs can be used to document and you can always keep that. [When] they are coming to assess how we have performed, I remain with that medical information. Then you can say how [you] did this, how you managed that, and then you refer them [to the records] and they assess the level of performance* ***#HM_5***

**Barriers**

***Data management requirements over stretch available human resources.***

It emerged from the study that the project came along with rigorous data requirements which were passed on to routine services within the health facilities upon donor exit. Despite absorption of project staff within public service, some departments like records did not experience the same. The resultant effect in the midterm and likely to stretch into the long term is that the data requirements far exceed the staff capacities especially in tertiary health facilities as echoed in the quote below;

*[we were elevated to] a hospital status here. We have two records officers, and now the system is very hectic. That means to sit on the computer all the time, then remember there is also HIV data which is much. Then general data which is also much. Then we can also get compromised quality because of overload [and yet we] can’t have the third records officer, [because] the system says two, what do we do now? do we get a volunteer? so, it is a challenge. Now there is COVID-19 data, now that is worse. We got those vaccines, we worked day and night and now people are looking for their certificates on line and they [records officers] have not finished entering the data in the system because we are having very few staff in the records.* ***#HM_2***

***Variations in data capture within the catchment area.***

During donor support, there were noted changes in data reporting arrangements where lower level facilities reported through other higher-level health facilities. Where as this was effectively implemented during donor support, health facilities experienced challenges and returned back to prior reporting arrangements before donor support. In some areas, health facilities outside the catchment were reported to have been added for reporting purposes and health workers would be made to conduct outreaches at facilities outside their catchment. This may have been driven by the desire to meet some targets which would have been difficult if focus had been put solely on the catchment area. When there were indications of drop in performance post transition, the previous implementing partner returned to support the health facilities to realize the targets as reported in the quote below;

*“they [implementing partner] have always returned to tell us about our first ANC [performance], and they used to fund some of our outreaches but [were not] consistent. They told us the way they were collecting their data then. For example, they felt that the people receiving services at health centre A [HCII] were supposed to be counted under facility B [General hospital]. But at the district, the data collected from facility B would be credited on facility B and data collected from facility A would be credited under facility A. So, they wanted us to go to facility A and do antenatal from there so that the data appears under facility B. [they shared the data] and there was a very big gap that time. That is when they tried to put in a lot of effort so that we hit their target, but still it was not achieved, it was too much because we did those outreaches in that [health facility A] but still we did not hit the target.* ***#HW_5***

**Governance**

***The calibre of district leadership***

Absorption of project staff into public service and continued implementation of best practices from the donor supported project varied across districts post-transition. To some extent, it was attributed to the calibre of leaders in both the political and technical wings of the district management. The political leadership in newly formed districts embraced the SMGL best practices more than the older peers. Much of the project capacity building was addressing an already felt health systems challenge which was contextually the much-needed external support at the time. New districts appreciated support as part of their health systems strengthening efforts and ambition. Others had more interest in responding to MCH which had been a bigger felt problem before the project, especially the more rural districts. Former project staff used to be nationally recruited indicating that some were posted to work in districts other than their origin. Leadership that was more receptive and open to recruitment into district health services such health workers who were originally from other districts witnessed higher absorption rates. Individual attributes also worked to support the continuity of service delivery post-transition, especially technical leadership. District Health Officers (DHOs) who were more proactive and responsive in terms of advocating for more staff recruitment to fill the gaps within the staffing norms oversaw the quick absorption of project staff into public service. The level of support during SMGL varied even among health facilities at the same level. This may have impacted the morale of managers and health workers post-transition which could have led to the observed performance. Rural Health Centre (HCIVs) for example seemed to have benefited more compared to urban Health Centre (HCIVs) especially those that were closer to the Regional Referral Hospital (RRH). As if that was not enough, the stability of health leadership could also have impacted the performance. One District Health Officer (DHO) from a maintained performance district started out as an SMGL project staff and would later get absorbed into public service and grew through the ranks to become the DHO. Another DHO had been with the same district before SMGL, participated in its implementation and continued to oversee the implementation of SMGL-recommended practices post-transition. Such institutional memory and stability of leadership may have contributed to the consistent implementation of recommendations.

**Barriers**

***Unstable district technical leadership***

Changes in the governance structures may have affected aspects of project promoted interventions such as accountability and reporting in terms of data management. Some urban health facilities would later be moved from the district oversight to city health authorities after one urban centre was elevated to city status. Still, in areas where health managers were in acting capacities affected decision making. These were reported to often shoulder undue influence from the political wing which would sometimes manifest through affected service delivery. For critical decisions perceived to attract resentment from the political wing, they would defer as reflected in the quote below;

*Leadership is very, very critical you find that in some districts they are very unstable. The other day [xxxx] was telling me we don’t have a substantive DHO. You don’t have someone there, you want a particular person but maybe he is still [in] school, whether [he] is there but you see he is not qualified. So, they have just put somebody there and that one also affects because there is no leadership. And when you are just there [in] acting [capacity] everybody will push you. If you get very [unlucky] and get a [bad] person, they will just push you. So, you [remain] there fearing and hesitant to take decisions.* **#HM_1**

**Medical products and supplies.**

***Support for medicines and supplies from successor grants***

Earlier mentioned of successor grants that followed observed that the bridged the gaps that were created. Some of these included provision of Blood Pressure [BP] machines, thermometers to ensure quality service provision. In one of such successor grants that followed, the implementing partner’s objective was to ensure sustenance of performance on selected indicators while responding to challenges that would deter mothers from utilizing the available health services. Health managers and health workers would be encouraged to use the RBF proceeds to purchase such supplies as gloves to ensure no scarcity of the same was witnessed.

*for them they wanted to support [and] to motivate the health workers but after you have done your work very well and have given them numbers and [delivered] quality service to the clients. And then also, stop these mothers from buying the supplies. When they came without gloves, the money they gave us we were supposed to get a percentage and buy supplies****. #HM_6***

**Barriers**

***Medicines and supplies stock-outs***

Immediately after the donor transition, there was a period of turbulence when interruptions in the supply of medicines and essential commodities were experienced at health facilities. This followed a switch from a donor-supported commodity supply chain to National Medical Stores. A common thread highlighting shortages in supplies emerged noting that health facilities were constrained with supplies and would oftentimes be forced to request patients to bring along supplies such as gloves to receive services;

*‘On another side also, we had enough supplies because the project was looking at human resources and at the same time it was looking at the supplies. If I can compare that time and today, a client comes and you have to ask, where are your gloves, when you don’t have them, ok, go and bring them for me gloves so that I can examine you. But the other time a mother would come, you say welcome, lay here, so that you examine, take history then you provide the services without asking for anything because things were available at the time.* ***#HW_3***

**Dynamic interaction across building blocks**

We observed that the applied framework pays minimal attention to the dynamic interactions across the building blocks beyond analysing each building block as a separate analytical entity. It holds that financing and governance building blocks for example may adversely impact on health workforce performance. This in itself can influence the outcomes as it relates to the continuity of MCH service delivery post donor transition. Taking on a system thinking perspective, service delivery which is the ultimate outcome of the interaction of all the other building blocks was reported to have experienced continuity albeit some challenges. There were instances from the health financing mechanisms which reflected more support to rural health facilities compared to their urban peers. This affected health workforce distribution and retention post transition. The consistent application of learning from project also reflects on the interactions across system building blocks. That is, improvements in the monitoring systems (such as MPDSR) may have greatly contributed to the ability for the system to have continuous quality improvement strategies for service delivery. Financing particularly for the recurrent budget together with medicines and supplies continues to experience recurring challenges with some arising from the additional mandates due to improvements in the other building blocks. Subnational level governance remains delicate and susceptible with the outcomes highly dependent on the personalities of office bearers at a particular point in time.

**Discussion**

We conducted this study to explore the impact of donor transition on the continuity of service provision for maternal and newborn health at the sub-national level in Uganda between 2012-2022. Our results reveal that overall, there was continuity of RMNCH service provision for most of the components introduced or reinforced by the project albeit some disruptions especially as it related to medicines and supplies stockouts, community component and the private sector component among others. Part of the reason to explain these observations was that implementation followed a phased approach which allowed for learning and to plough back the lessons into improving the design and delivery of RMNCH services that were responsive to the implementation context. The global commitment to addressing maternal and newborn mortality which remains strong and present as reflected in successor funding opportunities that continued post transition partly explained the continuity of MCH service provision. The role and availability of successor external grants from other donors, counterpart funding from the government during and after, absorption of the former project-supported workforce into public service, the continued use of infrastructure left behind, support for MCH services under PEPFAR grant post-transition cannot be ignored. The demand for available facility-based MCH services popularised during the project like NICU continued post-transition and contributed to the continuity of service provision. Our interpretation of these observations follows next.

A key finding from this study was the commitment from global actors to address maternal and newborn mortality which remains present in Uganda as reflected in the successor grants. The continuity in funding opportunities for MCH post donor transition partly explained the continuity of RMNCH service delivery. Some of the RNMCH indicators are a sensitive reflection of the health systems efficiency such as MMR, stillbirth, NMR and under-5 mortality. Alignment of funding to the same indicators within the successor grants could have played a role in continuity of service provision. In fact, within the Ugandan context, many of the RMNCH indicators are used as a measure of the quality and performance of the health systems which are reported routinely through the national DHIS2 data and surveillance systems. This has maintained performance momentum for both national and subnational indicators to ensure their optimal performance. Their political sensitivity culminated in the localised mechanism which compels both national and subnational political and technical leaders to provide oversight and close monitoring of performance. On various occasions, maternal deaths have led to uproar and a review of the processes before the occurrence. Uganda routinely compiles and submits reports on localised global commitments and national progress towards UHC and SDG 2030 targets. The continued commitment towards RMNCH even after some donors transition support has seen others move in to bridge the gaps. Globally this has remained true and, in some instances, has compelled the country to ensure they measure up to global requirements by committing and meeting their obligations(19, 20).

The role and availability of counterpart funding from government and continued support to MCH programs from other USAID projects could have played a great part in the maintenance of RMNCH service coverage post-transition. The United States Government through PEPFAR continued to support MCH service delivery post-transition. Unlike other donor-transitioned projects, the government demonstrated a unique commitment to counterpart funding for RMNCH services during and after donor exit. There was a noted increase in budgetary allocation towards MCH services. Funds were committed to upgrading lower-level health facilities to match service delivery requirements in form of infrastructure improvements. In addition, the government committed funds in form of staff salaries, and staffing norms revision which facilitated the absorption of former project staff into public service. Both the global and national commitments towards the improvement of RMNCH services could have been the drive behind different actors stepping up to address the gaps left behind by the SMGL exit hence providing continuity of service delivery which could have led to the maintenance of coverage. The ability to secure funding post-transition is a recognised form among sustainability strategies(21). This calls for national government commitment to secure counterpart funding to bridge gaps left behind after donor exit not only for MCH but also for other health system-wide interventions.

The demand for and availability of the facility-based MCH services popularised during the project could explain the continuity of service delivery post-transition. It emerged from the study that the newly introduced Neonatal Intensive Care Units (NICU) were very popular within the communities. Service users would nostalgically refer to them as the “clinics for small babies” which were initially not part of the MCH service delivery package before SMGL. Having learnt about the availability of such services, communities continued to use them post donor transition especially mothers with sick neonates a fact that may have contributed to the continuity of service delivery. This heavy investment in infrastructure in addition to rehabilitation and expansion of theatres and maternity wards is reflective of alignment of project features that strengthen the health systems and likely to be continued even after donor exit. This is perhaps a pointer to addressing health systems bottlenecks that often imped the demand within communities for both maternal and neonatal care services which must have been a felt need whose response was long overdue. It could also be that a certain level of behavioural change dose effectiveness towards the use of facility-based MCH services was achieved. Elsewhere studies have revealed that the nature of intervention contributes to its sustainability post-donor funding if it resonates well with the services users’ needs as it appears to have been the case in this context(21-23).

However, challenges to the maintenance of MCH service delivery post-donor transition were also observed. A key insight was the inability to meet the demand for medicines and supplies by the system post-transition. A common thread emerged of a resurgence of drug stockouts which were last experienced before the donor support. The reasons for this could be the nature of the national drug distribution system under National Medical Stores (NMS) which is centralised and sometimes overwhelmed with demand, logistical challenges and mismatches in procurement orders. It could also be explained by the complex drug delivery systems with a mix of the push for lower-level health facilities and the pull for tertiary-level facilities. Other reasons may to some extent hinge on the drug redistribution arrangements at a subnational level which requires authorisation hence occasioning delays in some instances. Perhaps the possible reason for this observation could be that drug procurement and distribution arrangements under donor support were set in project mode with minimal effort to prepare for transition into routine standards under public service drug distribution systems. Respondents noted that during donor support, the quick turnaround time to address impending stockouts was common with project support. This changed post-transition where stock-outs due to delayed or late delivery were observed. It could also be true that during donor support, demand for uptake of health services and consequently medicines and supplies increased for which the system was not prepared to support post-transition. With the whole-of-systems approach, perhaps the medicines and supplies building block was not accorded as similar attention as compared to human resources, governance and service delivery. This is typical of complex interventions where weaknesses from one component have the potential to affect aspects of the others as has been reported in a systematic review of evidence-based interventions(21).

Continuity in delivery of maternal and newborn service was affected by the inability to sustain the private sector component in absence of external financial support. Different aspects of private sector involvement such as the engagement of private maternal health service providers through reimbursing costs to deliveries conducted at their premises could not effectively continue post transition. This was especially the case given that being a rural setting, many mothers and their families could not afford certain services such as caesarean section when not subsidised. The other aspect of private sector involvement was through the private motorcycle riders to effect timely community referrals. Without voucher reimbursements post transition, securing their cooperation to continue transporting mothers to health facilities proved difficult. Whereas it was easy to secure their cooperation to evacuate pregnant women to the nearest health facilities for delivery in anticipation of transport refund, this aspect completely collapsed after the cessation of funding. The reason for this could have been that most riders were lay people who were not anchored in any ongoing government program the way Village Health Teams (VHTs) were and hence difficult to sustain post-transition. It could also be due to the context in which subdistrict health systems operate which is characterised by scarce resources with only PHC funds at their disposal which is often inadequate and mostly prioritised for addressing supply-side gaps and less demand-side challenges. The inability to easily secure successor funding for the private sector component was a major factor as has been reported elsewhere(21). Although the global terrain seems to reflect a shift from external donor support in favour of country ownership, perhaps working with the private sector is an area that has not received the due attention that it should. This is especially true given the recognition the private sector has received more recently in partnering to achieve Universal Health Coverage (UHC), a global campaign enshrined within the Sustainable Development Goals (SDG) targets. Whereas most donor support is directed towards the public and private-not-for-profit sector, there is a need for more engagement of the private-for-profit sector to widen coverage and increase access to quality maternal and child health services especially in lower income countries where these challenges still persist.

Within this study, we established that community level processes too either enabled or acted as barriers to continuity of MCH service delivery post donor transition. Through the application of the health systems building block as a guiding framework to understand the health systems response post donor transition, we have observed that none of the building block could adequately host these community level experiences. Perhaps this is the experience that other researchers have encountered when articulating community level health systems experiences while applying this framework. Given that the health systems building block framework is organised along inputs, processes and outcomes pathway, it is worth reflecting on the same as they relate to community level factors. In the era of Sustainable Development Goals (SDGs) and a drive towards UHC which aims to leave no one behind, the community health systems are increasingly gaining renewed attention. Researchers applying the health systems building blocks framework will most likely encounter similar limitations while analysing community level responses to UHC attainment. It is thus our considered observation that perhaps this is a great opportunity for the proponents of this framework to reflect on revisiting it in order to create favourable anchor points for community level experiences within this widely applied framework to critically appraise the health systems performance. The 1978 Alma Ata declaration shone a spotlight on the role of the community in health promotion and attainment of good health, the drive towards UHC provides a window to illuminate interest in streamlining the community level factors into the health systems building blocks framework.

**Limitations**

This study was not without limitations. The first is the recall bias which is attributed to studies adopting a longitudinal approach to data collection. The time lapse between key events and data collection may pose challenges where respondents cannot recall all relevant information as it pertained to those key events. This was addressed by aiding the respondents to reconstruct the key events guided by the objectives of the study. The exploratory inquiry technique also helped in offering prompts and signposts during the interview process which supported the recollection of events in whose information we were interested. Secondly, tracing some of the key respondents that would have been crucial to this study was another limitation. Many had moved on and we were constrained while tracing for them. However, we addressed this gap by attempting to review key project documents and retrieve relevant information about the implementation of the project. Thirdly, applied framework does not provide for opportunity to analyse the dynamic interactions across the building blocks beyond analysing results for each as a separate entity and yet the performance of one can have a spiralling effect on the rest of the building blocks. We addressed this by presenting the last paragraph of the results as the “dynamic interaction of the building blocks”. Lastly, the inability to anchor the demand side/community level experiences within any of the six building blocks of the applied framework was a limitation. We attempted to address this by identifying particular elements corresponding to a building block and presenting results and discussions implicitly under that building block. For example, because community referrals which had to do with service delivery and health financing, were presented and discussed under the service delivery and health financing building blocks and not as an independent community level factors. Nonetheless, caution is called for while interpreting these results where the context may limit their transferability. Despite the foregoing, we attempted to address all highlighted limitations to enhance data reliability.

**Conclusion**

Overall, a general perception of the continuity of maternal and newborn health service provision post-donor transition was observed with internal (government counterpart funding) and external enablers (successor donor funding) contributing to this performance. Opportunities for the continuity of maternal and newborn service delivery performance post-transition exists when harnessed well within the prevailing context such as political will to commit counterpart funding and recruitment of staff which can be even extended to strengthen the community component. The ability to learn and adapt, the presence of government counterpart funding and commitment to carry on with implementation were major ingredients signalling a crucial role of government in the continuity of service provision post-transition.

**List of abbreviation**

ANC Antenatal Care

CDC Centre for Disease Control and Prevention

CEmONIC Comprehensive Emergence, Obstetric and Newborn Intensive Care

DHMT District Health Management Teams

DHO District Health Officer

GHI Global Health Initiatives

HC Health Centre

MCH Maternal and Child Health

MDGs Millennium Development Goals

MMR Maternal Mortality ratio

MPDSR Maternal and Perinatal Death Surveillance and Response

NICU Neonatal Intensive Care Unit

PEPFAR Presidential Emergency Plan for AIDS Relief

PHC Primary Health Care

RBF Result-Based Financing

RMNCH Reproductive Maternal Newborn and Child Health

RRH Regional Referral Hospital

SDGs Sustainable Development Goals

SMGL Saving Mothers Giving Life

URMCHIP Uganda Reproductive Maternal and Child Health Improvement Project

USAID United States Agency for International Development

UHC Universal Health Coverage

**Declarations**

**Ethical approval and consent to participate**

This study received ethical approval from Makerere University School of Public Health Higher Degrees Research and Ethics Committee (HDREC) **ref; SPH 2021-128** and from the Ugandan National Council for Sciences and Technology (UNCST) **ref; HS2112ES.** Further administrative clearance was obtained from the management of all participating health facilities and administrative units where the study was conducted. Individual informed consent was obtained from all prospective respondents before interviews.

**Consent for publication**

Not applicable

**Availability of data and materials**

The datasets used and/or analysed during the current study are available from the corresponding author upon reasonable request.

**Competing interests**

The authors declare that they have no competing interests.

**Funding**

This study was supported by the Alliance for Health Policy and Systems Research (Alliance). For the full list of Alliance donors, please visit [*https://www.who.int/alliance-hpsr/partners/en/*](https://www.who.int/alliance-hpsr/partners/en/)*.* Any opinion, finding and conclusion or recommendation expressed in this material are that of the authors. The funder had no role in the design of the study, data collection, analysis, and interpretation of data and in writing the manuscript

**Author’s contribution**

ES, HZ, and FS conceived and designed the study. ES, and HZ conducted data collection. ES, and JN analysed the data and discussed the structure of the manuscript; ES wrote the first draft. ES, HZ, JN, AK, CK, TM and FS provided an interpretation of the study results. All authors reviewed and approved the final manuscript.

**Acknowledgements**

We wish to acknowledge the contribution of the data collection team, the management of the participating institutions, the respondents that provided invaluable information in this study and the anonymous reviewers who provided feedback.

**Author’s information**

***Affiliations***

**Department of Health Policy Planning and Management, School of Public Health, College of Health Sciences, Makerere University- Kampala, Uganda.**

*Eric Ssegujja, Justine Namakula, Henry Zakumumpa, Freddie Ssengooba*

**Department of Statistics and Population Studies, College of Business and Management Studies, Makerere University- Kampala, Uganda**

*Allen Kabagenyi*

**Ministry of Health- Kampala, Uganda**

*Carol Kyozira, Timothy Musila*

***Corresponding author***

Correspondences to Eric Ssegujja; [ssega2001@yahoo.com](mailto:ssega2001@yahoo.com)

**ORCID**

Eric Ssegujja <http://orcid.org/0000-0002-7732-1019>

Henry Zakumumpa: https://orcid.org/0000-0002-8169-1151

Justine Namakula: https://orcid.org/0000-0002-6784-2996

Allen Kabagenyi: https://orcid.org/0000-0002-2185-7535

Freddie Ssengooba: https://orcid.org/0000-0003-3489-0745

**References**

1. Bharali I, Mao W, Huffstetler H, Hoole A, Perera P, Ogbuoji O. Perspectives on transitions away from donor assistance for health: a discrete choices experiment in Sri Lanka. The Lancet Global Health. 2020;8:S25.

2. Sarriot E, Ricca J, Yourkavitch J, Ryan L. Taking the long view: a practical guide to sustainability planning and measurement in community-oriented health programming. Calverton: Macro International. 2008.

3. Scheirer MA, Dearing JW. An agenda for research on the sustainability of public health programs. American journal of public health. 2011;101(11):2059-67.

4. Healey J, Conlon CM, Malama K, Hobson R, Kaharuza F, Kekitiinwa A, et al. Sustainability and scale of the Saving Mothers, Giving Life approach in Uganda and Zambia. Global Health: Science and Practice. 2019;7(Supplement 1):S188-S206.

5. Goldberg J, Bryant M. Country ownership and capacity building: the next buzzwords in health systems strengthening or a truly new approach to development? BMC public health. 2012;12(1):1-9.

6. Oberth G, Whiteside A. What does sustainability mean in the HIV and AIDS response? African Journal of AIDS Research. 2016;15(1):35-43.

7. Sarriot EG, Swedberg EA, Ricca JG. Pro-sustainability choices and child deaths averted: from project experience to investment strategy. Health Policy and Planning. 2011;26(3):187-98.

8. Bennett S, Singh S, Ozawa S, Tran N, Kang J. Sustainability of donor programs: evaluating and informing the transition of a large HIV prevention program in India to local ownership. Global health action. 2011;4(1):7360.

9. Organization WH. Beginning with the end in mind: planning pilot projects and other programmatic research for successful scaling up. 2011.

10. Quam L, Achrekar A, Clay R. Saving Mothers, Giving Life: a systems approach to reducing maternal and perinatal deaths in Uganda and Zambia. Global Health: Science and Practice; 2019. p. S1-S5.

11. Serbanescu F, Clark TA, Goodwin MM, Nelson LJ, Boyd MA, Kekitiinwa AR, et al. Impact of the saving mothers, giving life approach on decreasing maternal and perinatal deaths in Uganda and Zambia. Global Health: Science and Practice. 2019;7(Supplement 1):S27-S47.

12. Palaia A, Spigel L, Cunningham M, Yang A, Hooks T, Ross S. Saving lives together: a qualitative evaluation of the Saving Mothers, Giving Life public-private partnership. Global Health: Science and Practice. 2019;7(Supplement 1):S123-S38.

13. Sensalire S, Isabirye P, Karamagi E, Byabagambi J, Rahimzai M, Calnan J. Saving mothers, giving life approach for strengthening health systems to reduce maternal and newborn deaths in 7 scale-up districts in northern Uganda. Global Health: Science and Practice. 2019;7(Supplement 1):S168-S87.

14. Rosenfield A, Maine D. Maternal mortality-a neglected tragedy: Where is the M in MCH? The Lancet. 1985;326(8446):83-5.

15. Bhutta ZA, Chopra M, Axelson H, Berman P, Boerma T, Bryce J, et al. Countdown to 2015 decade report (2000–10): taking stock of maternal, newborn, and child survival. The lancet. 2010;375(9730):2032-44.

16. Lomazzi M, Borisch B, Laaser U. The Millennium Development Goals: experiences, achievements and what's next. Global health action. 2014;7(1):23695.

17. Organization WH. Monitoring the building blocks of health systems: a handbook of indicators and their measurement strategies: World Health Organization; 2010.

18. Mulumba M, Basaza RK, Muhumuza FK. TRACKING THE PROGRESS AND IMPLICATIONS OF THE GLOBAL FINANCING FACILITY (IN THIS CASE RESULTS-BASED FINANCING) IN THE HEALTHCARE SECTOR IN UGANDA. 2021.

19. Shiffman J. Generating political priority for maternal mortality reduction in 5 developing countries. American journal of public health. 2007;97(5):796-803.

20. Shiffman J, Smith S. Generation of political priority for global health initiatives: a framework and case study of maternal mortality. The lancet. 2007;370(9595):1370-9.

21. Hailemariam M, Bustos T, Montgomery B, Barajas R, Evans LB, Drahota A. Evidence-based intervention sustainability strategies: a systematic review. Implementation Science. 2019;14(1):1-12.

22. Nyamtema AS, Urassa DP, van Roosmalen J. Maternal health interventions in resource limited countries: a systematic review of packages, impacts and factors for change. BMC pregnancy and childbirth. 2011;11(1):1-12.

23. Lassi ZS, Musavi NB, Maliqi B, Mansoor N, de Francisco A, Toure K, et al. Systematic review on human resources for health interventions to improve maternal health outcomes: evidence from low-and middle-income countries. Human resources for health. 2016;14(1):1-20.
