# Supplementary material for: The impact of donor transition on continuity of maternal and newborn health service delivery in Rwenzori sub-region of Uganda: a qualitative country case study analysis
Source: Global Health. 2023 Jul 10;19:48. doi: 10.1186/s12992-023-00945-6 (PMC10334577; doi:10.1186/s12992-023-00945-6)
Supplement: Supplementary file 3 — Supplementary Material 3 [file 12992_2023_945_MOESM3_ESM.docx]

The Editor

BMC Globalisation and Health

14^th^/04/2023

Sir/Madam,

Please find enclosed a manuscript titled, **“****The impact of donor transition on maternal and newborn health service delivery in Rwenzori sub-region of Uganda: A qualitative country case study analysis.”.** The manuscript has not been submitted anywhere.

The manuscript is a culmination of the research conducted to assess the impact of donor transition on maintenance of coverage of maternal and newborn health services. The transition of donor-supported health programmes to country ownership is gaining increasing attention due to reduced development assistance for health globally. It is further accelerated by the ineligibility of previously Low-Income Countries’ elevation into Middle-income status. Despite the increased attention, little is known about the long-term impact of this transition on the maintenance of coverage of maternal and child health services. To understand these dynamics, we conducted this study to explore the impact of donor transition on the maintenance of coverage of maternal and newborn health services at the sub-national level in Uganda between 2012-2021.

Results show that coverage of maternal and newborn health service provision was to a greater extent maintained post-donor support. The process was characterised by a phased implementation approach which may have given ample time to prepare the health systems for take-off post-transition. The pilot was conducted in districts perceived at the time to have had the highest burden of maternal mortality in the country. The embedded learning offered the opportunity to plough back lessons into intervention modification which reflected contextual adaptation. The availability of successor grants from other donors, counterpart funding from the government through absorption of USAID-project salaried workforce onto the public sector payroll, the continued use of infrastructure, and support for MCH services under PEPFAR support post-transition contributed to the maintenance of coverage.

Our team believes that this study is a timely contribution to the debates around the donor transition effects on health systems performance and how to situate governments to take over MCH service delivery after donors have ceased support.

Sincerely,


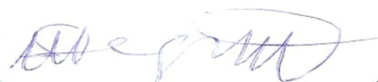


Eric Ssegujja

On behalf of the co-authors
