# Supplementary material for: The impact of donor transition on continuity of maternal and newborn health service delivery in Rwenzori sub-region of Uganda: a qualitative country case study analysis
Source: Global Health. 2023 Jul 10;19:48. doi: 10.1186/s12992-023-00945-6 (PMC10334577; doi:10.1186/s12992-023-00945-6)
Supplement: Supplementary file 2 — Supplementary Material 2 [file 12992_2023_945_MOESM2_ESM.docx]

**Reviewer comments**

**Submission ID:** GLAH-D-23-00182 R1

**Manuscript Title:** The impact of donor transition on continuity of maternal and newborn health service delivery in Rwenzori sub-region of Uganda: A qualitative country case study analysis.

We thank the Editor and the three reviewers for their thorough and valuable reviews. We have made amendments accordingly, and the recommendations have strengthened and promoted the quality of the manuscript. Please find submitted tracked and clean versions of the manuscript. A point-by-point list of changes is found below. Page and line numbers refer to the tracked version of the manuscript. Additional amendments related to the comments have been made in some places and are highlighted in tracked changes our responses. There are also some changes in the manuscript for clarity.

**Reviewer #1:**

**Comment:** The authors have responded to the comments and suggestions on the initial version of the manuscript with a re-structured and revised version of the paper. The revised version provides a clearer structure, while the reference to the 'continuity' of services provides a clearer focus for the analysis. Overall the revised version of the paper has addressed the issues and comments on the initial version and can be accepted for publication.

**Response:** we thank you very much for taking your time to read through the revised version of the manuscript. We also appreciate this comment supporting the publication of the revised version.

**Comment:** Table 2 and use of building blocks framework provides a clearer structure, and summarises the key findings through the division into enablers and barriers. However, it would be helpful to provide numbering for each of the building block sections, as it is not always clear where one building block concludes and another commences.

**Response:** This comment is well appreciated; indeed, we have made revisions to effect this recommendation. The building blocks have now each been assigned numbers within table 2 of the revised manuscript

**Page 7-8, Lines, 264-265:**

**Comment:** Responses to the specific recommendations on the initial version are listed below. Responses are satisfactory to all issues raised, with greater clarity through the consistent use of a single analytic framework.

**Response:** We appreciate you for this comment and observation. It is well received and appreciated.

**Reviewer #3:**

**Comment:** Thank you for your detailed response to reviewers. Below I have a few additional comments on the revised manuscript, particularly some points about explicitly noting the limitations of the building blocks framework (the omission of interactions across building blocks from a systems thinking perspective and the lack of explicit community voice -- which you address by including community engagement under service delivery, but would be good to explicitly mention that you are applying the building blocks framework while addressing its limitations).

**Response:** This observation is well received and appreciated. Revisions have been made to the different parts of the manuscript to incorporate this recommendation. Specifically, we have added a subsection as the last paragraph of the results where we present the interactions across the building blocks as reflected in the extracts below;

***“Interaction across building blocks***

*We observed that the applied framework pays little attention to the dynamic interactions across the building blocks beyond analysing each building block as a separate analytical entity. It holds that financing and governance building blocks for example may adversely impact on health workforce performance. This in itself can influence the outcomes as it relates to the continuity of MCH service delivery post donor transition. Taking on a systems thinking perspective, service delivery which is the ultimate outcome of the interaction of all the other building blocks was reported to have experienced continuity. This can be said to have been influenced by the project and government investments in infrastructure, equipment and relative improvements in health workforce numbers. Financing particularly the recurrent budget together with medicines and supplies continues to experience recurrent challenges with some arising from the increased mandates arising from improvements in the other building blocks. Governance remain susceptible with the outcomes highly dependent on individual personalities holding offices at a particular point in time”.*

**Page 18, Lines, 696-714:**

Further still, we have highlighted in our limitations the issue of the inability for the health systems building blocks framework to anchor the demand side/community level factors satisfactorily as reflected in the extract from the manuscript below;

**Page 22, Lines, 888-899:**

*“Thirdly, applied framework does not provide for opportunity to present the interactions across the building blocks beyond analysing results for each as a separate entity and yet the performance of one can have a spiralling effect on the rest of the building blocks. We addressed this by presenting the last paragraph of the results as the “dynamic interaction of the building blocks”. Lastly, the inability to anchor the demand side/community level experiences within any of the six building blocks of the applied framework was a limitation. We attempted to address this by identifying particular elements corresponding to a building block and presenting results and discussions under that building block. For example, because community referrals had to do with service delivery, we presented and discussed it under the service delivery building block and not an independent block say demand side or community factors”.*

**Comment:** Abstract: There is an open quote under methods before "health system building blocks." A note that the WHO's health system building blocks are not a complete enough theoretical framework on their own to guide thematic analysis, but are a useful way to structure findings. Would suggest using something like the following wording: "Thematic analysis was deductively conducted with findings structured by the WHO's health systems building blocks (Governance, Human resources, Health financing, Health information systems, medical products and supplies and service delivery) framework."

**Response:** This comment is particularly much appreciated. We have revised reflect this recommendation as reflected in the extract from the manuscript here below;

**Page 1, Lines, 30-34:**

*Thematic analysis was deductively conducted with findings structured along the WHO's health systems building blocks (Governance, Human resources, Health financing, Health information systems, medical products and supplies and service delivery) framework.*

**Comment:** I would further stress that when using the building blocks framework, it is necessary to note its shortcomings and to explore them in your analysis. These include a lack of focus on community needs (demand side factors) -- so in your paper, a note about community engagement in post-donor transition efforts -- as well as the interactions across building blocks and the ways these interactions affect outcomes. You have some of this implicitly in your paper, but it would be good to just note at the top that while the findings are separated by building block, attention has been paid to how interactions across building blocks and across different actors affect inputs, outputs, and outcomes of the system. I would suggest putting this note about the limitations of the building blocks framework and your attempt to ensure that you are capturing the community engagement aspect (noting that you've included it under service delivery) and the interactions across building block in the section called "Analytic framework" under "Methods."

**Response:** This comment is much appreciated, in fact it has reshaped the methods, results and discussion and limitation sections of this manuscript. We have made several revisions in those sections to reflect the recommendation as presented in several extracts below;

**Page 6, Lines, 222-224:**

*“It is however lean on articulating demand side/ community components and the dynamic interactions across the building blocks which have potential to impact the health systems performance”.*

As a way of introducing and situating the results, we guide readers on how the results are presented and how we incorporated aspects that the framework could not accommodate into the results;

**Page 7, Lines, 266-272:**

*“It is important to note that while the findings are separated by building blocks, attention has been paid to how interactions across building blocks and across different actors affect inputs, processes, outputs, and outcomes of the system which are implicitly reflected earlier on under the “analytical framework” within the methods section and later within the results (service delivery) and discussion sections that follow”.*

We also added a section as the last paragraph of the results to reflect on how the dynamic interactions across the building blocks could have impacted the continuity of MCH service delivery post donor transition;

**Page 18, Lines, 695-714:**

***“Interaction across building blocks***

*We observed that the applied framework pays little attention to the dynamic interactions across the building blocks beyond analysing each building block as a separate analytical entity. It holds that financing and governance building blocks for example may adversely impact on health workforce performance. This in itself can influence the outcomes as it relates to the continuity of MCH service delivery post donor transition. Taking on a systems thinking perspective, service delivery which is the ultimate outcome of the interaction of all the other building blocks was reported to have experienced continuity. There were instances from the health financing mechanisms which reflected more support rural health facilities compared to the urban peers. This affected health workforce distribution and retention post transition. The consistent application of learning from project also reflects on the interactions across system building blocks. That is, improvements in the monitoring systems (such as MPDSR) may have greatly contributed to the ability for the system to have continuous quality improvement strategies for service delivery. Financing particularly for the recurrent budget together with medicines and supplies continues to experience recurrent challenges with some arising from the increased mandates arising from improvements in the other building blocks. Governance remain susceptible with the outcomes highly dependent on individual personalities holding offices at a particular point in time”.*

Additionally, we reflected it as a limitation of the study and demonstrate how we made attempts to address this limitation;

**Page 22, Lines, 888-899:**

*Thirdly, applied framework does not provide for opportunity to analyse the dynamic interactions across the building blocks beyond analysing results for each as a separate entity and yet the performance of one can have a spiralling effect on the rest of the building blocks. We addressed this by presenting the last paragraph of the results as the “dynamic interaction of the building blocks”. Lastly, the inability to anchor the demand side/community level experiences within any of the six building blocks of the applied framework was a limitation. We attempted to address this by identifying particular elements corresponding to a building block and presenting results and discussions implicitly under that building block. For example, because community referrals which had to do with service delivery, were presented and discussed under the service delivery building block and not as an independent block say demand side or community factors.*

**Comment:** Table 2 is very helpful. Could consider splitting out an additional row for community engagement rather than having it under service delivery (at your discretion). Where relevant, it would be helpful to note how a finding in one building block.

**Response:** This comment is much appreciated and well received. However, having made revisions which show how community aspects have been incorporated within the service delivery building block and highlighting it as a limitation to the applied framework, we are unable to again treat it as a separate block within the summary table as suggested by the reviewers.

**Comment:** The barriers section under health financing is a bit weak. For example, not entirely clear what the "demand side component" here is. The sentence about investment in remote and rural health facilities, is a good opportunity to apply a systems thinking perspective to make note of potential unintended consequences of efforts to maintain post-transition performance and the interactions across building blocks (e.g. health financing mechanisms to support rural health affecting health workforce distribution and retention). The "Consistent application of learning from project." is another opportunity to make note of interactions across system building blocks. That is, making note of how monitoring systems (such as MPDSR) contributing to the ability to have continuous quality improvement strategies for service delivery.

**Response:** The comment is well received and appreciated. we have revised this particular section to make the statement clearer as reflected in the extract below;

**Page 10, Lines, 342-349:**

*“The inability to fund some components post-transition such as the community component was observed. More investments in remote and rural health facilities could have affected the morale and performance of the urban health facilities which did not receive the same package of support post-transition. The unintended consequence arising out of this was that for some of the lower level urban facilities that perceived themselves as having been supported less during donor support compared to their rural peers, the motivation to uphold and continue delivery of quality services varied”.*

We have further expanded on the last paragraph of the results section which reflects on the dynamic interactions across the building blocks to show how an action in one building block could have affected in another building block post donor transition as reflected in the extract below;

**Page 18, Lines, 701-712:**

*“Taking on a systems thinking perspective, service delivery which is the ultimate outcome of the interaction of all the other building blocks was reported to have experienced continuity. There were instances from the health financing mechanisms which reflected more support rural health facilities compared to the urban peers. This affected health workforce distribution and retention post transition. The consistent application of learning from project also reflects on the interactions across system building blocks. That is, improvements in the monitoring systems (such as MPDSR) may have greatly contributed to the ability for the system to have continuous quality improvement strategies for service delivery. Financing particularly for the recurrent budget together with medicines and supplies continues to experience recurrent challenges with some arising from the increased mandates arising from improvements in the other building blocks”.*

**Comment:** To drive your point home even more strongly in the conclusion, I would suggest including a parenthetical statement after "context" to include what exactly is meant by context (which aspects -- community needs? political will? health workforce training and accreditation? geography? etc).

**Response:**

**Page 22, Lines, 907-914:**

*“Opportunities for the continuity of maternal and newborn service delivery performance post-transition exists when harnessed well within the prevailing context such as political will to commit counterpart funding and recruitment of staff which can be even extended to strengthen the community component”.*
